# Molecular mechanisms involved in high glucose‐induced valve calcification in a 3D valve model with human valvular cells

**DOI:** 10.1111/jcmm.15277

**Published:** 2020-04-19

**Authors:** Mihaela Vadana, Sergiu Cecoltan, Letitia Ciortan, Razvan D. Macarie, Monica M. Tucureanu, Andreea C. Mihaila, Ionel Droc, Elena Butoi, Ileana Manduteanu

**Affiliations:** ^1^ Institute of Cellular Biology and Pathology ‘Nicolae Simionescu’, Biopathology and Therapy of Inflammation Bucharest Romania; ^2^ Cardiovascular Surgery Department Central Military Hospital Bucharest Romania

**Keywords:** calcific aortic valve disease, high glucose levels, human valvular cells, osteogenic molecules, valve tissue engineering

## Abstract

Calcific aortic valve disease (CAVD)—the most common valvular heart disease—is accelerated in diabetes and has no pharmacotherapy. Although it is known that early CAVD is associated with inflammation and osteogenesis, the molecular mechanisms involved in diabetes‐associated CAVD still need to be uncovered. In this context, we have developed a 3D construct based on gelatin populated with human valvular endothelial cells (VEC) and valvular interstitial cells (VIC) and evaluated the effect of high glucose (HG) concentration on osteogenic molecules expression and on calcification mechanisms. First, we characterized the 3D model and assessed VIC remodelling properties at different time‐points. Then, we exposed it to normal glucose (NG) or high glucose (HG) for 7, 14 and 21 days after which the cells were isolated, separated and investigated individually. Our results showed that encapsulated VIC actively remodel the hydrogel, as demonstrated by an increased expression of extracellular matrix (ECM) proteins and matrix metalloproteinases (MMPs). Moreover, exposure of the construct to HG triggered bone morphogenetic protein (BMP) and TGF‐β signalling pathways, up‐regulating expression of osteogenic molecules—BMP‐2/‐4, osteocalcin, osteopontin, SMADs and Runt‐related transcription factor (Runx‐2)—and increased calcium deposits in an osteogenic environment. These findings underline the potential of the developed 3D model as a suitable system to investigate the mechanisms of human CAVD and may help to better understand the calcification mechanisms in CAVD associated to diabetes.

## 1 INTRODUCTION

Aortic valve (AV) stenosis due to calcific aortic valve disease (CAVD) is the most common heart valve disease in developed countries.^1^ About 33% of elderly patients have echo‐cardiographic evidence of calcific aortic valve sclerosis—an early and subclinical form of CAVD.^2^, ^3^ The presence of CAVD is very common in patients with type 2 diabetes (occurring in up to ~45% of patients) and is associated with an increased risk of degenerative aortic valve disease and of cardiovascular mortality, independently of established risk factors.^4^, ^5^

Currently, pharmacological approaches for slowing down the progression of this disease are unavailable, with surgical repair and valve replacement as the primary treatment of CAVD.^6^ Some of the reasons responsible for the absence of proper pharmacotherapy include (a) the lack of a proper valve leaflet model where valvular cells might communicate better and (b) the molecular pathways through which valvular calcification is triggered still need to be uncovered.

The human AV is an avascular tri‐leaflet structure with a thickness of approximately 1 mm, comprised of an outer layer of valve endothelial cells (VEC), three internal layers—fibrosa, spongiosa and ventricularis—made up in majority of valve interstitial cells (VIC) and different matrix elements. VEC maintain valve homeostasis by regulating permeability, inflammatory cell adhesion and paracrine signalling with interstitial cells.^7^ VIC are a group of heterogeneous cells with high plasticity and mechanical adaptability regulated both biomechanically and biochemically.^8^ They are similar to fibroblasts when quiescent and to myofibroblasts when activated, their phenotype being modulated by spatial variations in matrix elasticity and organization.^9^, ^10^ Differentiation of VIC from fibroblast to myofibroblast phenotype is critical for wound healing in vivo and may also be critical in the production of native extracellular matrix (ECM) during valve tissue engineering.^11^ VIC actively maintain and repair heart valve tissue by producing ECM, including collagen, elastin and glycosaminoglycans, that provide tensile strength and elasticity to the valve.^12^ However, in diseased valves, the persistent activation of VIC disrupts normal cellular and matrix architecture and can lead to calcification. The role of VIC in the progression of CAVD continues to be a subject of intense research, although their implication was demonstrated by experiments that showed the transition of VIC to osteoblasts and calcific nodules formation.^13^

Previously, it was shown that interactions between VIC with specific peptides of ECM promote specific cellular functions and subsequently the calcification process.^14^ It was also found that maintenance of a quiescent phenotype of VIC is highly sensitive to culture conditions. Therefore, when cultured in conventional culture (2D), VIC undergo spontaneous activation, similar to pathological differentiation, which intrinsically limits their use for in vitro models to study CAVD evolution.^15^ When VIC were cultured on soft hydrogel substrates, the expression level of many critical genes was restored to level measured in freshly isolated cells.^16^ More interesting data came from co‐culture experiments between VIC and VEC, where it was demonstrated that calcific nodule formation was inhibited by a nitric oxide donor,^17^ implying the beneficial role of VEC. Moreover, in a study on VIC‐VEC communication, porcine VEC prevented VIC from calcification in osteogenic medium, reflected in normal expression of osteogenic molecules Runt‐related transcription factor (Runx2) and osteocalcin (OSC).^18^

We recently found that AV function and structure was affected in a hyperlipaemic ApoE^‐^/^‐^ diabetic mouse model even from the first week of diabetes onset, due to effects on remodelling and osteogenic molecules.^19^ However, insufficient data exist regarding the effect of high glucose (HG) levels associated with diabetic conditions, on VEC/VIC phenotype in the context of valvular calcification. Previously, our group showed that HG induces enhanced monocyte adhesion to VEC via a mechanism involving the cell adhesion molecules: ICAM‐1, VCAM‐1 and CD 18.^20^ Recently, using a 2D culture, it was found that VIC from sheep origin do not show morphological changes and do not acquire an osteogenic phenotype in hyperinsulinaemia or hyperglycaemia.^21^

In this context, a 3D scaffold with human VEC and VIC, that supports and promotes VIC‐mediated ECM remodelling, while preserving VIC fibroblastic phenotype, would be promising for the study of valvular cells interaction in normal as well as pathological conditions, and molecular mechanisms of leaflet calcification. Therefore, we developed and characterized a 3D construct with human valvular cells, exposed it to HG concentration for 7, 14 and 21 days and investigated the effect of HG on main osteogenic molecules and signalling mechanisms associated with valvular calcification. As previous data showed that VIC grown in gelatin‐based hydrogels exhibited key characteristics of their native morphology,^10^ we used a similar hydrogel composition.

Our findings indicate that, in the first two weeks, VIC dynamically remodel the hydrogel microenvironment by deposition of native ECM and synthesis of MMPs (MMP‐1, MMP‐2, MMP‐9 and MMP‐13). After 2 weeks, VIC exhibit a quiescent phenotype with low levels of α‐SMA, while VEC are well differentiated and protect the 3D construct from calcification upon exposure to osteogenic conditions. When 3D constructs were exposed to HG conditions, VEC and VIC exhibit activated BMP‐2 and TGF‐β signalling pathways with increased expression of osteogenic molecules. These molecules were expressed differently in VIC versus VEC, suggesting both similar and different mechanisms of HG effects on valvular cells.

## 2 MATERIALS AND METHODS

### 2.1 Human VEC and VIC isolation and culture

Primary VEC and VIC were harvested from non‐calcified cusps (or portions of the cusp) of human aortic valves obtained from 3 patients who underwent valve replacement surgery (according to *Dr Carol Davila Central Military Emergency University Hospital* protocol) for severe calcific aortic valve stenosis. The investigation was carried out according to the principles outlined in the Declaration of Helsinki for experiments involving human samples.^22^ Participants gave their written informed consent by signing the appropriate paperwork and respecting their anonymity and privacy rights. The Ethics Committee of the Institute of Cellular Biology and Pathology ‘Nicolae Simionescu’ has approved the study.

**FIGURE 1:**
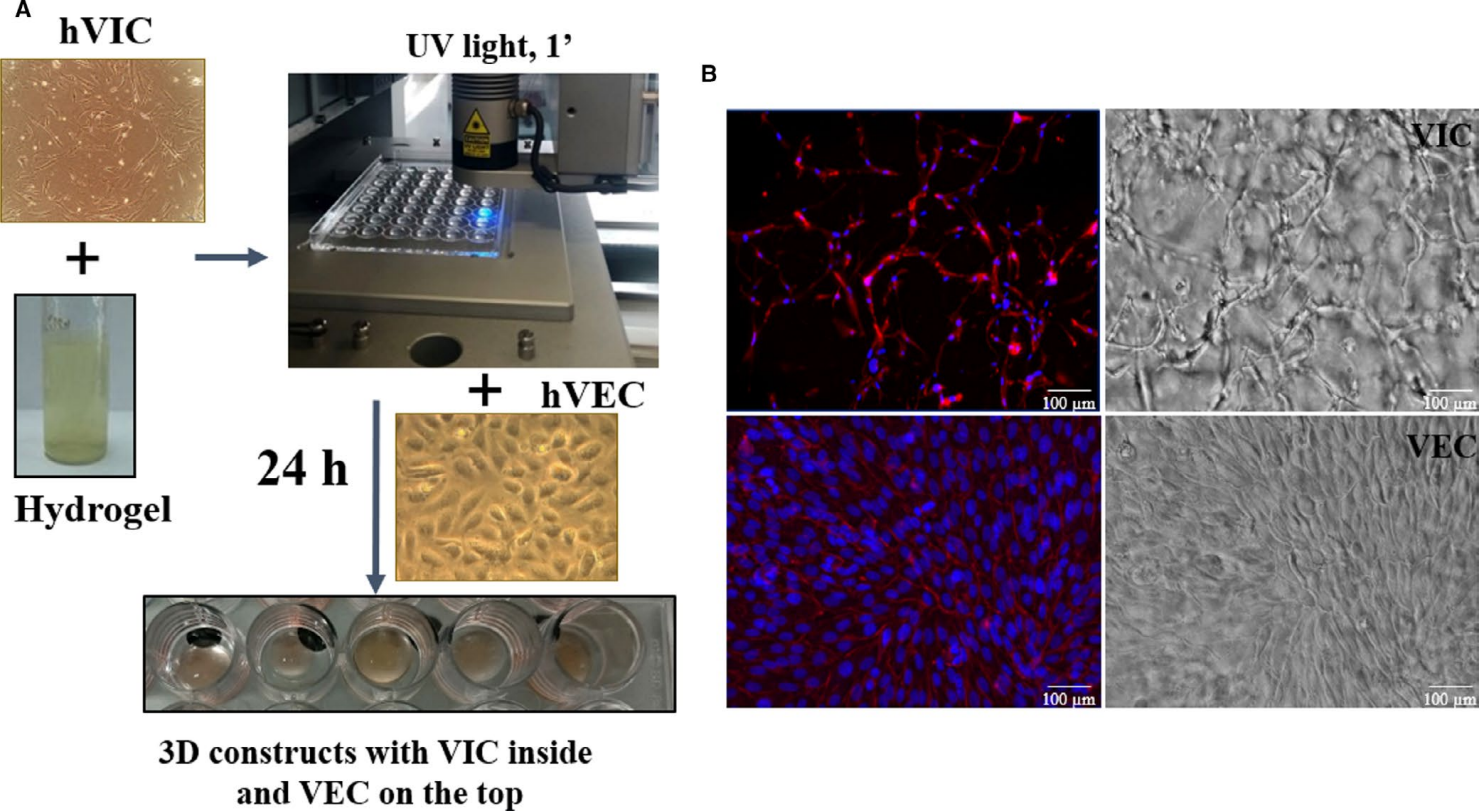
Obtaining the 3D construct, morphology of valvular cells from the 3D construct. A, 3D‐construct development: human VIC at a density of 2 million cells/mL were re‐suspended in G‐MA hydrogel solution (10% porcine gelatin methacrylate, 1% alginate and 0.5% Photo‐initiator—Irgacure 2595). 100 μL of VIC‐laden pre‐polymer solution was dropwise added on a 48‐cavity mould (Ø ‐ 8, 1 mm thickness) and subsequently cross‐linked by exposure for 1 minute to UV light (365 nm). Resulted 3D constructs were removed from the mould and cultured, according to protocol. After 24 h, VEC (5 × 10^5^/cm^2^) were seeded on top of 3D constructs. B, Valvular cells morphology in 3D construct at day 7 of culture as determined by phalloidin labelled F‐actin (red) and DAPI nuclear staining (blue). Hydrogel formula supports cell network development of VIC (inside the hydrogel) and VEC proliferation as a monolayer on the scaffold surface. Contrast phase images of the surface and inside of hydrogel with VIC encapsulated inside and VEC cultured on top. Scale bar indicates 100 µm

Calcific deposits or thickened lesions were removed from the leaflet, and the remaining non‐calcified tissue was cut into smaller pieces that were enzymatically digested for 5‐10 minutes with collagenase I (Biochrom/Merck) at 37°C. Released VEC were cultured in endothelial cell growth medium with 20% FBS (Gibco) and 100 U/mL penicillin, 100 μg/mL streptomycin and 50 μg/mL neomycin (Sigma‐Aldrich, Germany). VEC used in this work were isolated from three different patients (characterized in Table S1). For VIC isolation, enzymatic digestion of valve pieces was continued for 4‐5h at 37°C, using Liberase (Roche, Sigma). The resulted VIC were cultured in DMEM with 15% FBS and antibiotics. To limit the inter‐individual variability, we performed the majority of experiments using commercial VIC (cryo‐preserved valvular Interstitial cells/ P10462—Innoprot), except for VIC used in Figure 1B and Figure S1B, that were obtained from a single patient.

Following the first cell passage, CD31‐positive VEC were purified using magnetic beads conjugated to monoclonal anti‐human CD31 antibody (Miltenyi Biotec). VEC phenotype was confirmed by contact inhibited growth and expression of endothelial‐specific markers such as PECAM1 (CD31) and von Willebrand factor (vWF). VIC phenotype was established by expression of vimentin and alpha‐smooth muscle actin (α‐SMA).

### 2.2 Hydrogel synthesis

To obtain gelatin methacrylate used for hydrogel, we have adapted a previously described protocol.^23^

Briefly, powdered type A gelatin from porcine skin (Sigma‐Aldrich) was dissolved in phosphate buffer and heated (40°C) to obtain a 10 wt% gelatin solution. Gelatin methacrylate (G‐MA) solution was formed by adding dropwise 0.04 mL:1 g gelatin methacrylic anhydride under vigorous stirring for 1 hour at 45°C. The reaction between gelatin and methacrylic anhydride was inactivated by lowering pH to 4.5, using 1 mol/L HCl.

The G‐MA solution was then dialysed and frozen. Subsequently, the solution was lyophilized for 72 hours which yielded a white porous foam‐like material, which was stored at −30°C before experimental use.

Hydrogel solution was obtained by mixing 10% G‐MA and 1% sodium alginate in PBS. Alginate was added as a sacrificial polymer, that is rapidly released in culture, giving the hydrogel porosity, useful for cell feeding and oxygenation. Finally, 0.1%. of photo‐initiator (Irgacure 2595) was added to the mix.

### 2.3 3D constructs with VIC encapsulated in hydrogel and VEC seeded on top

Confluent VIC were trypsinized and mixed in the pre‐polymer solution (2 × 10^6^ cells/mL). 100 μL of cell‐laden pre‐polymer solution was dropwise added on a 48‐cavity mould (Ø ‐ 8 mm, 1 mm thickness, Figure 1) and subsequently cross‐linked by exposure to UV light (365 nm) for 1 minute. Constructs were removed from the mould and placed into a 48‐well plate containing DMEM with 10% FBS and antibiotics. After 24 hours, human VEC (50 000/cm^2^) were seeded on the constructs. For some experiments, constructs were exposed to osteogenic medium (OM) (control medium supplemented with 10 mmol/L β‐glycerophosphate, 10 ng/mL ascorbic acid and 10^−8 ^mol/L dexamethasone). Cells grown on tissue culture polystyrene (TCPS) were used as 2D control.

### 2.4 Cell morphology and distribution in the 3D constructs

Constructs were fixed with paraformaldehyde 4% for 4 hours and then permeated with 0.2% Triton X‐100 for 10 minutes. Phalloidin‐TRITC was used for immunofluorescent staining of cytoskeletal proteins and 4′,6‐diamidino‐2‐phenylindole (DAPI) for nuclear staining. Samples were visualized with a fluorescence microscope (Olympus IX81) and analysed using imaging software cellSens and ImageJ.

### 2.5 Western blot

Protein expression of alpha‐SMA, vimentin, SMAD 1/5/8/9, SMAD2/3, BMP‐2, Runx‐2 and actin/GAPDH was assessed in lysate of VIC cultured in 2D or VEC and VIC isolated and separated from 3D constructs. For separation, 3D constructs (10 pooled constructs/ sample) were exposed to 520U/ml Liberase, and VIC population was obtained by negative selection using endothelial marker CD31‐MicroBeads (Miltenyi Biotech). The separated cells were subjected to electrophoresis and immunoblot assay as previously described.^24^ SuperSignal West Pico (Pierce) was used as chemiluminescent substrate, and the signal was visualized and acquired using a LAS‐3000 (FujiFilm) Imaging System.

### 2.6 Quantitative RT‐PCR

Gene expression of matrix proteins (collagen I, III, elastin, laminin and fibronectin) and metalloproteases (MMP‐1, MMP‐2, MMP‐9 and MMP‐13) was evaluated using qPCR. For each experiment, cells from 10 to 12 constructs were pooled to isolate enough RNA for cDNA synthesis. RNA isolation was performed using PureLink RNA mini Kit (Ambion^™^, Carlsbad, CA). First‐strand cDNA synthesis was performed employing 1μg of total RNA and MMLV reverse transcriptase (Invitrogen). Assessment of gene expression was done by amplification of cDNA using a LightCycler 480 RT‐PCR System (Roche) and SYBR Green I chemistry. The primer sequences are shown in Table 1. The relative quantification was done by comparative CT method and expressed as arbitrary units. Beta‐actin was used as reporter gene for all the investigated molecules.

**Table 1:** The sequences of oligonucleotide primers used for evaluation of gene expression

Gene	GenBank^® ^accession number	Sequences of oligonucleotide primers	Predicted size (bp)
Coll I	NM_000089	Fw: 5’‐aattggagctgttggtaacgc‐3’ Rv: 5’‐caccagtaaggccgtttgc‐3’	125
Coll III	NM_000090.3	Fw: 5’‐aggtcctgcgggtaacact‐3’ Rv: 5’‐actttcacccttgacaccctg‐3’	226
Elastin	NM_000501.4	Fw: 5’‐cattcctacttacggggttgga‐3’ Rv: 5’‐ctccgacactagggacacc‐3’	95
Laminin	NM_005559.4	Fw: 5’‐gtgatggcaacagcgcaaa‐3’ Rv: 5’‐gacccagtgatattctctccca‐3’	116
MMP‐1	NM_002421.3	Fw:5’‐aaaattacacgccagatttgcc‐3’ Rv: 5’‐ggtgtgacattactccagagttg‐3’	82
MMP‐2	NM_004530	Fw: 5’‐ccgtcgcccatcatcaagtt‐3’ Rv: 5’‐ctgtctggggcagtccaaag‐3’	169
MMP‐9	NM_004994	Fw: 5’‐gtgcgtcttccccttcactttcct‐3’ Rv: 5’‐ggaatgatctaagcccagcg‐3’	199
MMP‐13	NM_002427.3	Fw: 5’‐actgagaggctccgagaaatg‐3’ Rv: 5’‐gaaccccgcatcttggctt‐3’	103
BMP‐2	NM_001200.4	Fw: 5’‐actaccagaaacgagtgggaa‐3’ Rv: 5’‐gcatctgttctcggaaaacct‐3’	113
BMP‐4	NM_130850.5	Fw: 5’‐aaagtcgccgagattcaggg‐3’ Rv: 5’‐gacggcactcttgctaggc −3’	135
Osteocalcin	NM_199173.6	Fw: 5’‐atgagagccctcacactcctc‐3’ Rv: 5’‐gccgtagaagcgccgatag‐3’	294
Osteopontin	NM_001040058.2	Fw: 5’‐gaagtttcgcagacctgacat‐3’ Rv: 5’‐gtatgcaccattcaactcctcg‐3’	91
RUNX2	NM_001024630.4	Fw: 5’‐ccgcctcagtgatttagggc‐3’ Rv: 5’‐gggtctgtaatctgactctgtcc‐3’	132
β‐actin	NM_001101.4	Fw: 5’‐catgtacgttgctatccaggc‐3’ Rv: 5’‐ctccttaatgtcacgcacgat‐3’	250

### 2.7 Immunofluorescence analysis

Cryosections from 3D constructs were incubated overnight at 4°C with specific antibodies. After incubation, the sections were washed and incubated with FITC‐conjugated or AlexaFluor594‐conjugated secondary antibodies for 1 hour, at RT. Nuclei were counterstained with DAPI. Stained sections were visualized under a fluorescence microscope. Secondary antibodies (antimouse, anti‐rabbit or anti‐goat fluorochrome‐coupled antibodies) were used as negative controls to evaluate specific staining of each primary antibody.

### 2.8 Alizarin Red assay

To quantify calcified matrix developed in normal or osteogenic conditions, 3D constructs were fixed with paraformaldehyde (4%, 1‐2 hours), washed three times with PBS and then incubated with 40 nmol/L Alizarin Red S. This dye binds to calcium crystals in cells or matrix fibres, revealing a red colour. Bound dye was released from the gels using 10% acetic acid. The concentration of dye in solution was then quantified using absorbance spectrophotometry at 405 nm wavelength.

### 2.9 Statistical analysis

For every type of method, we performed 3 different experiments—every experiment consisting of different number of constructs with VIC and VEC from passage 4‐7. Statistical analysis was performed with GraphPad Prism 7.0 with data points denoting mean ± standard deviation (SD). Statistical significance is shown as *P*‐values obtained via a two‐tailed Student's t test when comparing two experimental groups and analysis of variance (one‐way ANOVA) with multiple comparisons when comparing more than two groups. A *P*‐value of *P* < .05 was considered statistically significant.

## 3 RESULTS

### 3.1 3D‐construct development and characterisation

#### 3.1.1 Characterisation of human VEC and VIC

To characterize VEC and VIC isolated from human aortic valves, we assessed the expression of von Willebrand factor (vWF) and PECAM‐1 in VEC and the expression of vimentin and α‐SMA in VIC, using immunofluorescence technique. As shown, aortic VEC stained positive for endothelial markers vWF and PECAM‐1 (Figure S1A) and VIC were positive for α‐SMA and vimentin protein (Figure S1B).

#### 3.1.2 The morphology and viability of valvular cells from 3D constructs

After cell characterization, isolated VIC were encapsulated in G‐MA hydrogel and cross‐linked by exposure to UV light (Figure 1A). Subsequently, VEC were seeded on top, and resulted 3D constructs were cultured for different time periods. During the first week, VIC exhibited a fibroblast‐like phenotype, became elongated and interconnected, forming complex cell networks as shown in Figure 1B (at 7 days). VEC seeded on top proliferate and form a monolayer over the hydrogel as shown in the phase contrast picture or TRITC‐phalloidin staining (Figure 1B). Moreover, immunofluorescence results performed on cryosections revealed that specific VEC markers are localized mainly on the surface, while VIC markers, fibroblast specific protein‐1 (FSP‐1) and vimentin, are located inside the construct (Figure S2). The viability of cells from constructs was investigated at 7, 14 and 21 days. Approximately 98% of cells remained viable after 7 days, whereas at 14 and 21 days, the number of cells exceeded the number of cells initially encapsulated per construct (Figure S1C). These results suggest that cells from constructs are viable and are able to proliferate.

#### 3.1.3 Phenotype of encapsulated VIC

**FIGURE 2:**
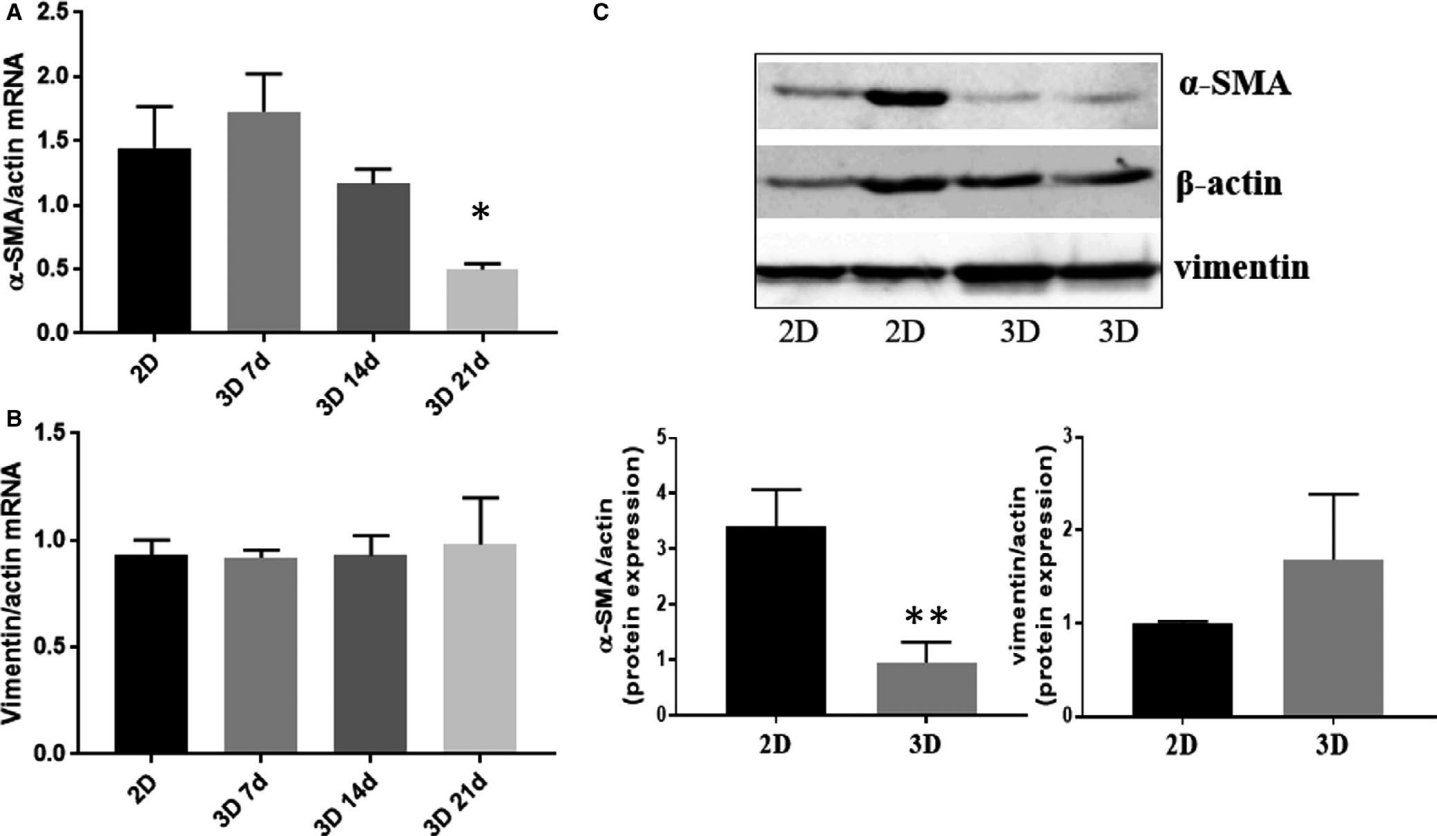
Phenotype of VIC and VEC from the 3D constructs. A and B, mRNA expression of VIC markers, α‐SMA (A) and vimentin (B), in VIC isolated from 3D constructs cultured for different period of time (7, 14 or 21 days), compared with VIC grown in 2D culture (7 days). n = 3, *P < .05, VIC from 3D compared with VIC from 2D. Data depicted as mean ± SD. C, Evaluation of protein expression for α‐SMA and vimentin in VIC isolated from 3D constructs or from 2D culture by Western blot, n = 3, *P < .05, VIC from 3D compared with VIC from 2D

Experiments done to investigate the phenotype of VIC revealed that the gene expression of vimentin was unmodified in time (Figure 2B), while that of α‐SMA started to decrease after 2 weeks in 3D constructs compared with 2D culture (Figure 2A). The gene expression decrease was reflected in a dramatic reduction of α‐SMA protein expression in encapsulated VIC from 3D constructs compared with VIC cultured in 2D (after 14 days), while vimentin expression remains unchanged (Figure 2C).

### 3.2 **VIC encapsulated in 3D** **constructs exhibit remodelling activity**

**FIGURE 3:**
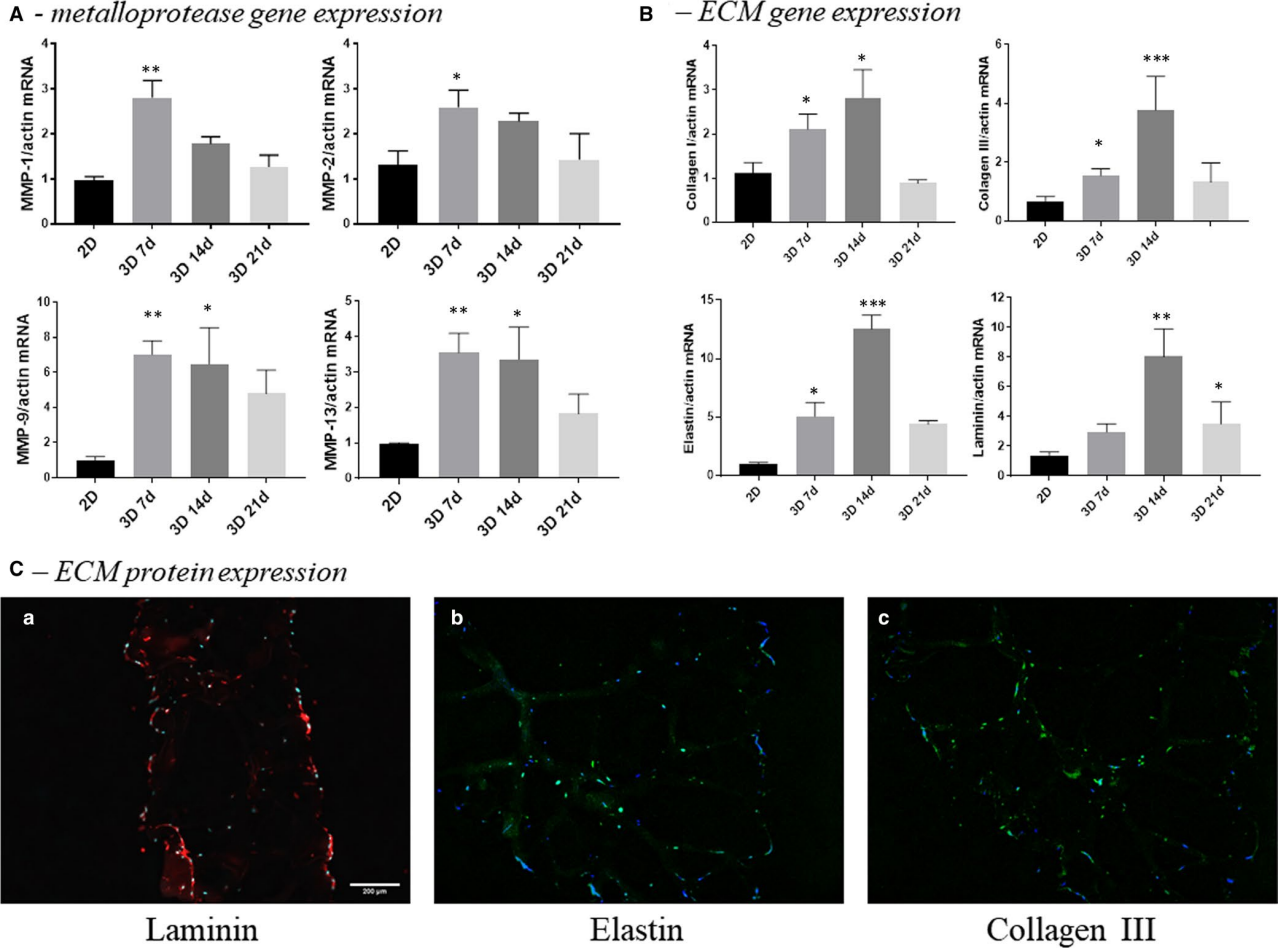
Expression of molecules associated with matrix remodelling by VIC cultured in 2‐dimensional conditions compared with VIC isolated from 3D constructs. A, Gene expression of matrix metalloproteases MMP‐1, MMP‐2, MMP‐9 and MMP‐13 in VIC cultured in 2D (7 days) versus VIC from 3D constructs at different periods of time—7, 14 or 21 days, as evaluated by real‐time PCR. B, Gene expression of extracellular matrix proteins by VIC cultured in 2‐dimensional conditions compared with VIC isolated from 3D constructs. The mRNA of matrix proteins was normalized to actin mRNA. Note that VIC isolated from 3D constructs exhibited a time‐dependent increase of collagen I, III, elastin and laminin mRNAs. n = 3, *P < .05, **P < .01, ***P < .001 VIC from 2D versus VIC from 3D. C, Protein expression of ECM as evaluated by immunofluorescent technique. Representative images of laminin (a), elastin (b) and collagen III (c) in sections obtained from 3D constructs with human VEC and VIC. The sections were marked with specific primary antibodies and Alexa‐ or FITC‐coupled secondary antibodies (red or green staining). Nuclei were stained with DAPI (blue staining)

VIC from 3D constructs exhibited a continuous expansion, a process that requires hydrogel remodelling, being dependent on both MMP activity and ECM production. Thereby, to investigate the capacity of encapsulated VIC for hydrogel remodelling, we analysed the expression of main MMPs and ECM proteins found in the aortic valve. After 7 days, we found enhanced gene expression of collagenases MMP‐1 and MMP‐13 and gelatinases MMP‐2 and MMP‐9 (Figure 3A) in encapsulated VIC compared with VIC grown in 2D. As shown in Figure 3A, after this time period, MMP expression starts to decrease, reaching control expression levels at 21 days. Interestingly, ECM components of valvular tissue—collagen I, collagen III, elastin and laminin—reach the maximum gene expression at day 14, followed by an important decrease at 21 (Figure 3B). Protein expression of ECM molecules was also confirmed by immunofluorescence assay on sections obtained from 3D constructs (Figure 3C).

### 3.3 The osteogenic environment activates encapsulated VIC and induces calcific nodule formation in 3D constructs

**FIGURE 4:**
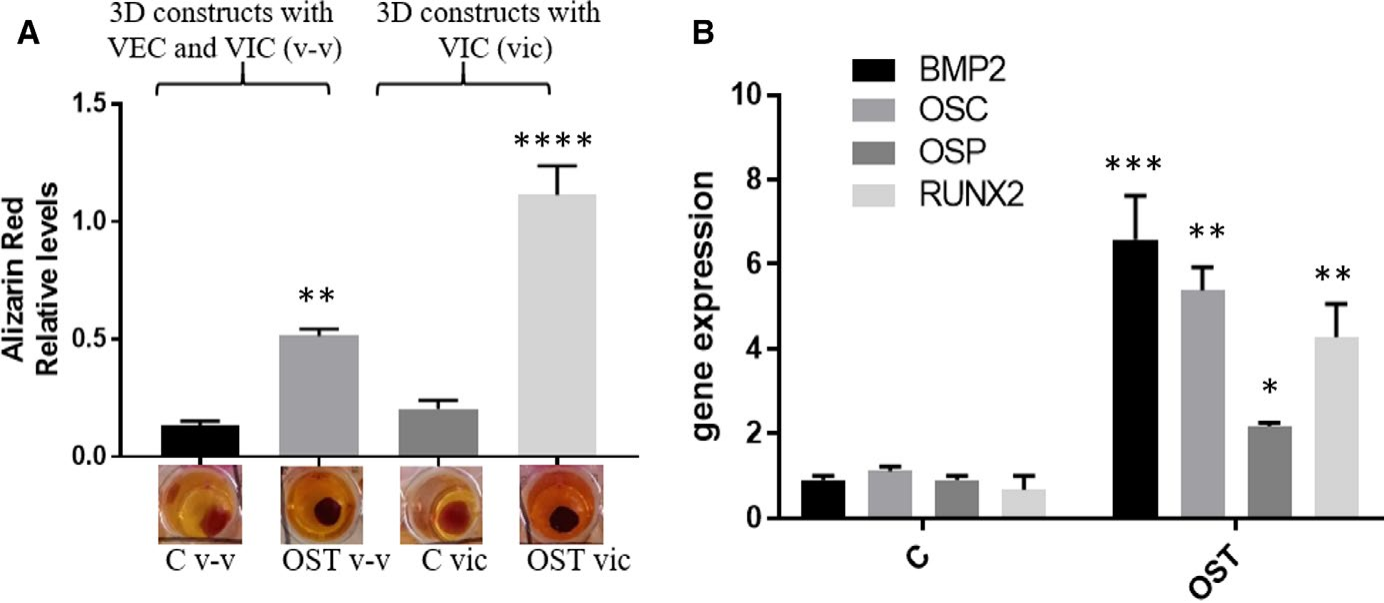
Osteogenic environment favours the calcium deposits development and induces osteogenic phenotype of VIC in 3D constructs. A, Alizarin Red was used to stain the mineralized nodules formed by cells from 3D constructs with VIC or 3D constructs with VEC and VIC (v‐v) cultured for 14 days. The lower panel shows the representative stained construct from each of the experimental group. The upper graph displays the quantitative measurement of Alizarin Red dye released from the mineralized nodules formed in 3D constructs cultured in normal conditions or exposed to osteogenic media (OST: 10 mM β‐glycerophosphate, 10 ng/mL ascorbic acid and 10^−8^ mol/l dexamethasone). n = 3, **P < .01, ****P < .0001. B, mRNA expression of osteogenic molecules (BMP‐2, osteocalcin, osteopontin and RUNX2) in VIC isolated from 3D constructs cultured in normal conditions or exposed to OST media for 14 days. Note that VIC isolated from 3D constructs exposed to OST media exhibit a significantly increased expression of osteogenic molecules mRNAs. n = 3, *P < .01, **P < .01, ***P < .001 VIC from 3D‐OST versus VIC from 3D. Data depicted as mean ± SD

In order to test the functionality of our 3D model to study the mechanisms of calcific valve diseases, constructs with VIC or with VEC‐VIC were exposed to osteogenic stimuli for 14 days. In normal culture media, constructs exhibited low reactivity for Alizarin Red staining, as show in Figure 4A. Contrary, exposure to osteogenic environment stimulated calcific nodule formation. Moreover, osteogenic media induced significantly higher (twofold increase) calcium deposits in constructs only with VIC, as compared to those with VIC‐VEC (Figure 4A), suggesting a protective effect of VEC over VIC osteogenic transformation. To further investigate the calcific process, we assessed osteogenic transformation of VIC. We found that BMP‐2 exhibited a 7‐time increased level in VIC from constructs exposed to osteogenic conditions compared with VIC from constructs grown in normal conditions (Figure 4B). Moreover, Runx2, osteopontin and osteocalcin mRNA were also significantly increased in VIC from osteogenic conditions.

### 3.4 High glucose concentration induces different expression of osteogenic molecules in VEC versus VIC from 3D construct

**FIGURE 5:**
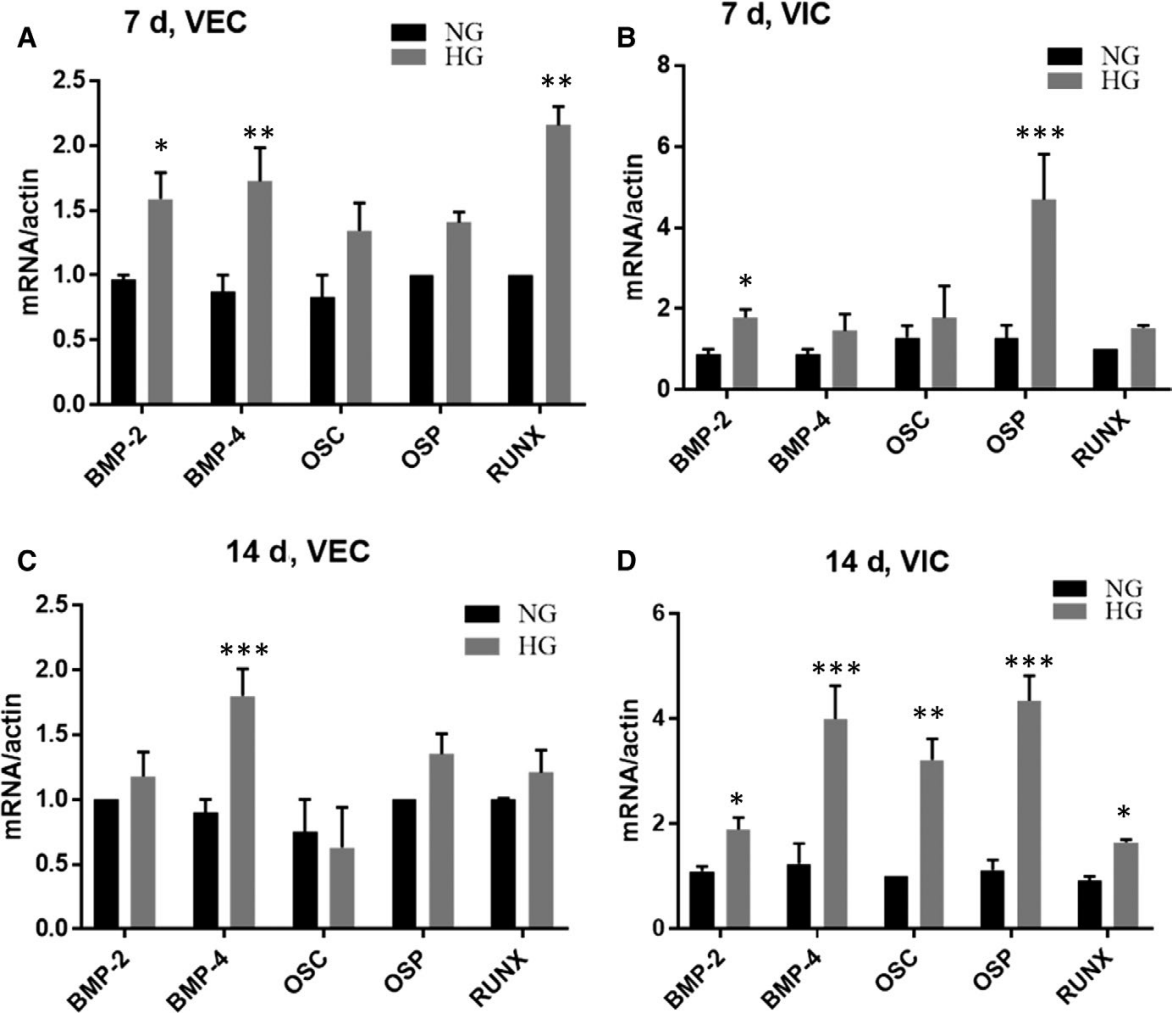
Gene expression of osteogenic molecules expressed by VEC and VIC from 3D constructs cultured in NG or HG conditions. A and C, Gene expression of BMP‐2, BMP‐4, osteocalcin, osteopontin and Runx‐2 in VEC isolated from 3D constructs after 7 (A) and 14 (C) days, as evaluated by real‐time PCR. n = 3. B and D, Gene expression of osteogenic molecules in VIC isolated from 3D constructs after 7 (B) and 14 (D) days, as evaluated by real‐time PCR. The mRNA of osteogenic molecules was normalized to actin mRNA. n = 3, *P < .05, **P < .01, ***P < .001 VIC from 2D versus VIC from 3D

Compared with non‐diabetics, patients with diabetes have greater calcification and increased expression of osteogenic molecules.^25^ Thus, to investigate whether and how the valvular cells respond to HG, 3D constructs were exposed to normal glucose (NG, 5 mmol/L) or HG (25 mmol/L) for 7 and 14 days. Molecules associated with the process of valve calcification were evaluated in VEC and VIC isolated and separated from 3D constructs. After 7 days, BMP‐2, BMP‐4 and Runx‐2 gene expressions were significantly increased by HG in VEC, while BMP‐2, osteopontin and Runx‐2 were significantly increased in VIC (Figure 5A, B). After 14 days, only BMP‐4 was still increased in VEC, while in VIC all the investigated osteogenic molecules were significantly increased by HG (Figure 5C, D).

### 3.5 TGF‐β and BMP‐2 signalling pathways are triggered by high glucose levels

TGF‐β and BMP signalling pathways are involved in different cellular processes, including bone formation during mammalian development and valve calcification. Signalling transduction by TGF‐β and BMPs occurs both, by canonical SMAD‐dependent pathways and non‐canonical SMAD‐independent signalling pathways (eg. p38 MAPK pathway),^26^ and converge at the Runx2 to control osteoblast‐specific genes expression.

However, it is not yet known whether BMP and TGF‐β pathways are activated by HG in human valvular cells. We supposed that a HG microenvironment could trigger these signalling pathways in valvular cells incorporated in our 3D model, process that could be responsible for valve calcification in diabetic patients.

**FIGURE 6:**
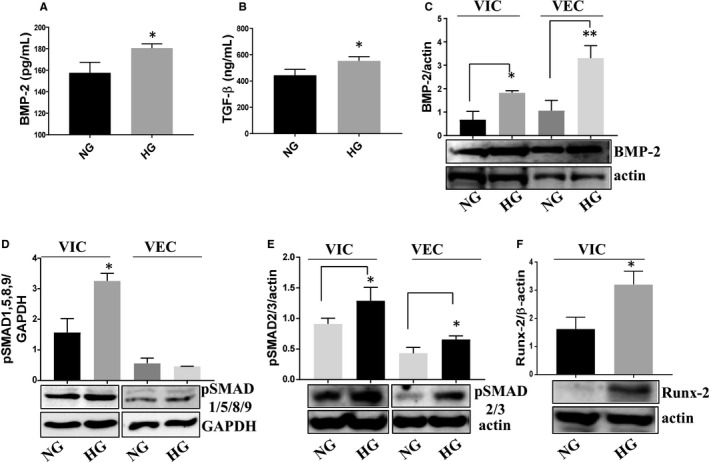
Protein expression of osteogenic signalling pathway molecules expressed by VEC and VIC from 3D constructs, cultured in NG or HG conditions. A and B, Soluble BMP‐2 and TGF‐β released in the conditioned media by valvular cells from 3D construct exposed to NG or HG conditions, as determined by ELISA assay. n = 3, *P < .05. C‐F, Protein expression of BMP‐2, pSMAD1/5/8/9, pSMAD2/3 and Runx‐2 in VIC and VEC as determined by Western blot. n = 3, *P < .05**P < .01 high glucose vs normal glucose

BMP‐2 and TGF‐β secretion was assessed by ELISA assay in media from VIC‐VEC 3D constructs exposed to NG or HG for 7 days. As shown in Figure 6A, B, HG significantly enhanced BMP‐2 and TGF‐β secretion. In addition, BMP‐2 protein expression was also found increased by HG in cell lysates of both VEC and VIC (Figure 6C). As SMADs are canonical mediators of TGF‐β and BMP signalling, we next investigated the activation of SMAD proteins induced by HG. Western blot analysis revealed that HG significantly induced phosphorylation of SMAD1/5/8/9 in VIC (Figure 6D) and of SMAD2/3 in both VEC and VIC (Figure 6E). As a consequence, protein expression of Runx‐2 was significantly increased in VIC isolated form 3D constructs exposed to HG for 7 days (Figure 6F).

### 3.6 Exposure of 3D constructs to osteogenic media increases calcium deposits in HG, compared with NG condition

**FIGURE 7:**
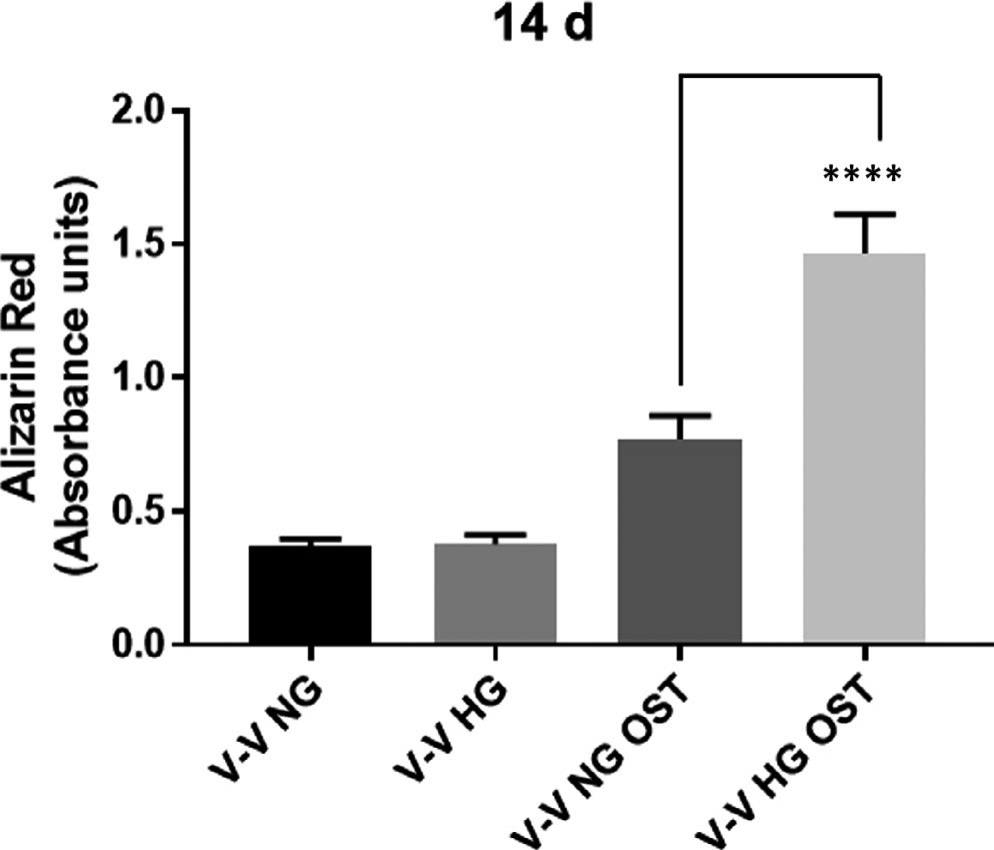
Calcium deposits development in 3D constructs exposed to NG or HG concentration in an osteogenic environment. Alizarin Red was used to stain the mineralized nodules formed by cells from 3D constructs with VEC and VIC cultured for 14 days in NG or HG in presence or absence of osteogenic media (OST). n = 3, ****P < .0001

As we have found that HG levels up‐regulate some key osteogenic molecules in VEC and VIC, we next investigated whether HG accelerates the calcification process. Therefore, constructs were exposed to NG or HG in the presence or absence of osteogenic stimuli for 14 days and then evaluated for calcium deposits. Samples exposed only to HG did not develop increased calcium deposits compared with NG. However, exposure to osteogenic environment in the presence of HG stimulated calcific nodule formation, as revealed by significant increase of calcium staining (Figure 7).

## 4 DISCUSSION

Calcific aortic valve disease is a degenerative disease characterized by inflammation, fibrosis and calcification, leading to hardening and stiffening of the aortic valve leaflet, processes that can cause death.^27^, ^28^ Although remarkable efforts have been made recently in heart valve tissue engineering, knowledge of valve cell biology, specifically of VIC, remains incomplete. The future of aortic valve research is likely to elucidate the mechanisms underlying the leaflet calcification, which could lead to new biomarkers and possible new targets for therapy. This will require the valve leaflet models to be as close as possible to the human leaflet and respond in a similar way as a normal valve to pathological and physiological conditions.

In this study we developed a 3D model for aortic valve leaflet and exposed it to HG concentration, in order to investigate the mechanism of CAVD in a diabetic environment. We have demonstrated that VIC encapsulated in this hydrogel gain a quiescent fibroblast‐like phenotype with low α‐SMA expression that spread and develop cell networks. Exposure to osteogenic conditions leads to the development of calcium deposits, suggesting that this model can be used to study the calcification process of the valve. Therefore, when constructs were exposed to HG concentration, both VEC and VIC developed an osteoblast‐like phenotype, with increased expression of osteogenic molecules belonging to TGF‐β and BMP‐2 signalling pathways.

Previously, different hydrogel formulas with natural or synthetic polymers have been used to create 3D scaffolds, in order to obtain a quiescent phenotype of VIC in vitro. Thus, culturing bovine VIC on hyaluronic acid‐based hydrogels exhibited stability over time, yielded a viability greater than 75% over 14 days but affected cell spreading, keeping them in a spherical shape.^29^ Conversely, human VIC loaded in collagen gels with various densities exhibited different cell viability and proliferation, with a maximum effect on VIC proliferation being observed for 1% collagen gel; however, these VIC exhibit increased expression of α‐SMA, possibly reflecting myofibroblastic differentiation.^30^ VIC encapsulated in hybrid hydrogels fabricated from hyaluronic acid and gelatin presented a quiescent phenotype, but exhibited a spherical morphology and did not develop cell networks, even though the concentration of encapsulated VIC was 10 million/mL.^31^

In our model, loaded VIC exhibited fibroblast‐like morphology and a dynamic phenotype, characterized by increased production of ECM proteins and MMPs, providing the basis for hydrogel remodelling. In the first week, VIC exhibited an activated phenotype characterized by enhanced expression of α‐SMA, MMPs and ECM proteins. In the second week, MMP production along with α‐SMA expression started to decrease, suggesting that VIC shift towards a quiescent phenotype. The decreased expression of both MMPs and ECM proteins found at 21 days may indicate that the hydrogel remodelling was diminished at this time, and VIC acquired a quiescent phenotype.

Although there is evidence that VIC may transform into myofibroblasts and osteoblasts in 3D gelatin‐based models,^31^ previous studies did not include endothelial cells. Our data revealed that 3D constructs only with VIC exhibited a significant increase in calcium staining compared with 3D constructs with VEC/VIC. These results suggest a functional communication between valvular cells and a protective effect of VEC on VIC transformation towards osteogenic phenotype.

It is known that diabetes is predictive of poor prognosis in valve disease and of faster degeneration for implanted bio‐prosthetic aortic valves,^32^, ^33^ but the molecular mechanisms involved in diabetes‐associated CAVD are unknown. In this study, we demonstrated that exposure of 3D constructs to diabetic conditions, led to increased expression of osteogenic molecules in valvular cells. Thus, expression of BMP‐2—the earliest and most specific maker of the osteoblastic phenotype—and of TGF‐β—also a mediator of aortic VIC calcification—was found increased in cells and in conditioned media from 3D constructs exposed to HG. Previously, it was found that HG levels induce TGF‐β in smooth muscle cells (SMC), dependent by PKC.^34^ As PKC is a protein kinase activated by reactive oxygen species (ROS), we performed experiments to evaluate whether ROS are increased by HG. Our preliminary data indicate an increased ROS expression in cells from constructs exposed to HG, (Figure S3). Therefore, we suppose that HG increases the TGF‐β and BMP‐2 dependent by ROS signalling pathway.

Subsequent to TGF‐β/BMP induction, both SMAD and p38 MAPK pathways converge to Runx2 and control osteoblast differentiation.^26^ Therefore, under HG conditions, both VEC and VIC from 3D construct exhibited significantly increased levels of phosphorylated form of SMAD2/3 with SMAD1/5/8/9 and of Runx‐2 increased only in VIC. The activated SMADs signalling pathways in VIC lead to overexpression of all investigated osteogenic molecules after 14 days. These results are in accord with our recent data demonstrating the increased protein expression of BMP‐2/‐4, OSC and OSP early, in a diabetic mouse model.^19^ The increased expression of osteogenic molecules induced by HG is reflected in elevated calcium deposits when the 3D constructs were exposed to an osteogenic environment.

There are few studies regarding the effects of HG on osteogenic pathways and molecules associated with aortic valve calcification. Recently, osteopontin gene expression and calcium deposition were not found modified by HG in VIC cultured in 2D system,^21^ differences that underline VIC response to 3D culture.

Other studies that investigated the effect of HG on vascular calcification found that HG increase the expression of Runx‐2 and BMP‐2 and calcification of vascular SMC.^25^ TGF‐β was also found important in vascular calcification in a study showing that TGF‐β1 induces osteogenesis in SMC, working synergistically with elastin degradation products.^35^ Moreover, HG coupled with elastin degradation products and TGF‐β cause a greater degree of osteogenesis to SMC by overexpression of typical osteogenic markers.^36^ These data, along with our results underline the involvement of TGF‐β and BMP‐2 signalling pathways in the osteogenic process associated to HG levels.

Our study is limited by the static nature of experiments. Blood flow–induced shear stress and hemodynamic forces have major roles in valve tissue development and in normal and pathological structural integrity of the valves.^37^ However, as biomechanical signals are transduced in interactions between VEC and VIC and ECM that modulate valve dynamic response,^38^ and our model allows cell‐to‐cell and ECM communication, we propose it as an appropriate model that can be used to further understand the role of valvular cells in CAVD initiation and progression.

Taken together, our data highlight the potential of 3D model with human valvular cells as a suitable system to investigate VIC phenotype changes, as a result of both communication with valvular endothelial cells and exposure to pathological stimuli. In addition, increased osteogenic molecules and calcium deposition induced by high glucose levels, dependent of TGF‐β and BMP‐2 signalling pathways, could explain the increased risk of degenerative aortic valve disease and calcification found in diabetic patients.

## CONFLICT OF INTEREST

The authors confirm that there are no conflicts of interest.

## AUTHOR CONTRIBUTIONS

MV performed the Western blot, immunofluorescence and ROS experiments. SC prepared the hydrogel and 3D constructs and performed phenotype analysis of cells. LC and RDM produced 3D constructs and performed qPCR experiments and English language revision. MMT isolated valvular cells and performed data analysis. ACM performed qPCR and calcium staining. ID operated and provided the aortic valves samples. EB and IM supervised the experiment(s), analysed the data and prepared the manuscript.

## Supporting information

Supplementary Material

## Data Availability

The data that support the findings of this study are available from the corresponding author upon reasonable request.

## References

[1] Benjamin EJ , Blaha MJ , Chiuve SE , et al. Heart disease and stroke statistics’ 2017 update: A report from the American heart association. Circulation. 2017;135:e146‐e603. 28122885 10.1161/CIR.0000000000000485 PMC5408160

[2] Otto CM , Prendergast B . Aortic‐valve stenosis‐from patients at risk to severe valve obstruction. N Engl J Med. 2014;21:744‐756. 10.1056/NEJMra1313875 25140960

[3] Thaden JJ , Nkomo VT , Enriquez‐Sarano M . The global burden of aortic stenosis. Prog Cardiovasc Dis. 2014;56:565‐571. 24838132 10.1016/j.pcad.2014.02.006

[4] Rossi A , Targher G , Zoppini G , et al. Aortic and mitral annular calcifications are predictive of all‐cause and cardiovascular mortality in patients with type 2 diabetes. Diabetes Care. 2012;35:1781‐1786. 22699285 10.2337/dc12-0134 PMC3402245

[5] Ginter E , Simko V . Type 2 diabetes mellitus, pandemic in 21st century. Adv Exp Med Biol. 2012;771:42‐50. 23393670 10.1007/978-1-4614-5441-0_6

[6] Lindman BR , Bonow RO , Otto CM . Current management of calcific aortic stenosis. Circ Res. 2013;113:223‐237. 23833296 10.1161/CIRCRESAHA.111.300084 PMC4013234

[7] Sacks MS , Yoganathan AP . Heart valve function: A Biomechanical perspective. Philos Trans R Soc Lond B Biol Sci. 2007;362:1369‐1391. 17588873 10.1098/rstb.2007.2122 PMC2440402

[8] Wyss K , Yip CY , Mirzaei Z , et al. The elastic properties of valve interstitial cells undergoing pathological differentiation. J Biomech. 2012;45:882‐887. 22189247 10.1016/j.jbiomech.2011.11.030

[9] Ma H , Killaars AR , DelRio FW , et al. Myofibroblastic activation of valvular interstitial cells is modulated by spatial variations in matrix elasticity and its organization. Biomaterials. 2017;131:131‐144. 28390245 10.1016/j.biomaterials.2017.03.040 PMC5452973

[10] Benton JA , DeForest CA , Vivekanandan V , Anseth KS . Photocrosslinking of gelatin macromers to synthesize porous hydrogels that promote valvular interstitial cell function. Tissue Eng Part A. 2009;15:3221‐3230. 19374488 10.1089/ten.tea.2008.0545 PMC2783792

[11] Durbin AD , Gotlieb AI . Advances towards understanding heart valve response to in injury. Cardiovasc Pathol. 2002;11:69‐77. 11934597 10.1016/s1054-8807(01)00109-0

[12] Masters KS , Shah DN , Walker G , et al. Designing scaffolds for valvular interstitial cells: cell adhesion and function on naturally derived materials. J Biomed Mater Res A. 2004;71:172‐180. 15368267 10.1002/jbm.a.30149

[13] Yip CY , Chen JH , Zhao R , Simmons CA . Calcification by valve interstitial cells is regulated by the stiffness of the extracellular matrix. Arterioscler Thromb Vasc Biol. 2009;29:936‐942. 19304575 10.1161/ATVBAHA.108.182394

[14] Gu X , Masters KS . Regulation of valvular interstitial cell calcification by adhesive peptide sequences. J Biomed Mater Res A. 2010;93:1620‐1630. 20073077 10.1002/jbm.a.32660 PMC2860685

[15] Benton JA , Fairbanks BD , Anseth KS . Characterization of valvular interstitial cell function in three dimensional matrix metalloproteinase degradable PEG hydrogels. Biomaterials. 2009;30:6593‐6603. 19747725 10.1016/j.biomaterials.2009.08.031 PMC2772097

[16] Wang H , Tibbitt MW , Langer SJ , et al. Hydrogels preserve native phenotypes of valvular fibroblasts through an elasticity‐regulated PI3K/AKT pathway. Proc Natl Acad Sci U S A. 2013;110:19336‐19341. 24218588 10.1073/pnas.1306369110 PMC3845151

[17] Kennedy JA , Hua X , Mishra K , et al. Inhibition of calcifying nodule formation in cultured porcine aortic valve cells by nitric oxide donors. Eur J Pharmacol. 2009;602:28‐35. 19056377 10.1016/j.ejphar.2008.11.029

[18] Richards J , El‐Hamamsy I , Chen S , et al. Side‐specific endothelial‐dependent regulation of aortic valve calcification: interplay of hemodynamics and nitric oxide signaling. Am J Pathol. 2013;182:1922‐1931. 23499458 10.1016/j.ajpath.2013.01.037 PMC3644712

[19] Tucureanu MM , Filippi A , Alexandru N , et al. Diabetes‐induced early molecular and functional changes in aortic heart valves in a murine model of atherosclerosis. Diab Vasc Dis Res. 2019;16:562‐576. 31530180 10.1177/1479164119874469 PMC6787765

[20] Manduteanu I , Voinea M , Serban G , Simionescu M . High glucose induces enhanced monocyte adhesion to valvular endothelial cells via a mechanism involving ICAM‐1, VCAM‐1 and CD18. Endothelium. 1999;6:315‐324. 10475094 10.3109/10623329909078498

[21] Selig JI , Ouwens DM , Raschke S , et al. Impact of hyperinsulinemia and hyperglycemia on valvular interstitial cells ‐ A link between aortic heart valve degeneration and type 2 diabetes. Biochim Biophys Acta Mol Basis Dis. 2019;1865:2526‐2537. 31152868 10.1016/j.bbadis.2019.05.019

[22] World Medical Association Declaration of Helsinki . Recommendations guiding physicians in biomedical research involving human subjects. Cardiovasc Res. 1997;35:2‐3. 9302340

[23] Shirahama H , Lee BH , Tan LP , Cho NJ . Precise tuning of facile one‐pot gelatin Methacryloyl (GelMA) synthesis. Sci Rep. 2016;6:31036. 27503340 10.1038/srep31036 PMC4977492

[24] Butoi E , Gan AM , Tucureanu MM , et al. Cross‐talk between macrophages and smooth muscle cells impairs collagen and metalloprotease synthesis and promotes angiogenesis. Biochim Biophys Acta. 2016;1863:1568‐1578. 27060293 10.1016/j.bbamcr.2016.04.001

[25] Chen NX , Duan D , O'Neill KD , Moe SM . High glucose increases the expression of Cbfa1 and BMP‐2 and enhances the calcification of vascular smooth muscle cells. Nephrol Dial Transplant. 2006;21:3435‐3442. 17005530 10.1093/ndt/gfl429

[26] Chen G , Deng C , Li YP . TGF‐β and BMP Signaling in Osteoblast Differentiation and Bone Formation. Int J Biol Sci. 2012;8:272‐288. 22298955 10.7150/ijbs.2929 PMC3269610

[27] Freeman RV , Otto CM . Spectrum of calcific aortic valve disease: Pathogenesis, disease progression, and treatment strategies. Circulation. 2005;111:3316‐3326. 15967862 10.1161/CIRCULATIONAHA.104.486738

[28] Xu S , Grande‐Allen KJ . The role of cell biology and leaflet remodeling in the progression of heart valve disease. Methodist Debakey Cardiovasc J. 2010;6:2‐7. 10.14797/mdcj-6-1-2 20360651

[29] Duan B , Hockaday LA , Kapetanovic E , et al. Stiffness and adhesivity control aortic valve interstitial cell behaviour within hyaluronic acid‐based hydrogels. Acta Biomater. 2013;9:7640‐7650. 23648571 10.1016/j.actbio.2013.04.050 PMC3700637

[30] Dreger SA , Thomas P , Sachlos E , et al. Potential for synthesis and degradation of extracellular matrix proteins by valve interstitial cells seeded onto collagen scaffolds. Tissue Eng. 2006;12:2533‐2540. 16995786 10.1089/ten.2006.12.2533

[31] Hjortnaes J , Goettsch C , Hutcheson JD , et al. Simulation of early calcific aortic valve disease in a 3D platform: A role for myofibroblast differentiation. J Mol Cell Cardiol. 2016;94:13‐20. 26996755 10.1016/j.yjmcc.2016.03.004 PMC4906202

[32] Katz R , Budoff MJ , Takasu J , et al. Relationship of metabolic syndrome with incident aortic valve calcium and aortic valve calcium progression: the Multi‐Ethnic Study of Atherosclerosis (MESA). Diabetes. 2009;58:813‐819. 19136658 10.2337/db08-1515 PMC2661576

[33] Lorusso R , Gelsomino S , Lucà F , et al. Type 2 diabetes mellitus is associated with faster degeneration of bioprosthetic valve: results from a propensity score‐matched Italian multicenter study. Circulation. 2012;125:604‐614. 22203696 10.1161/CIRCULATIONAHA.111.025064

[34] Lindschau C , Quass P , Menne J , et al. Glucose‐induced TGF‐beta1 and TGF‐beta receptor‐1 expression in vascular smooth muscle cells is mediated by protein kinase C‐alpha. Hypertension. 2003;42:335‐341. 12939231 10.1161/01.HYP.0000087839.72582.DD

[35] Simionescu A , Philips K , Vyavahare N . Elastin‐derived peptides and TGF‐beta1 induce osteogenic responses in smooth muscle cells. Biochem Biophys Res Commun. 2005;334:524‐532. 16005428 10.1016/j.bbrc.2005.06.119

[36] Sinha A , Vyavahare NR . High‐glucose levels and elastin degradation products accelerate osteogenesis in vascular smooth muscle cells. Diab Vasc Dis Res. 2013;10:410‐419. 23754846 10.1177/1479164113485101 PMC5403374

[37] Balachandran K , Sucosky P , Yoganathan AP . Hemodynamics and mechanobiology of aortic valve inflammation and calcification. Int J Inflam. 2011;2011:263870. 21760982 10.4061/2011/263870 PMC3133012

[38] Chen JH , Simmons CA . Cell‐matrix interactions in the pathobiology of calcific aortic valve disease: critical roles for matricellular, matricrine, and matrix mechanics cues. Circ Res. 2011;108:1510‐1524. 21659654 10.1161/CIRCRESAHA.110.234237

