# Supplementary material for: Molecular mechanisms involved in high glucose‐induced valve calcification in a 3D valve model with human valvular cells
Source: J Cell Mol Med. 2020 Apr 19;24(11):6350–61. doi: 10.1111/jcmm.15277 (PMC7294117; doi:10.1111/jcmm.15277)
Supplement: Supplementary file 1 — Supplementary Material [file JCMM-24-6350-s001.docx]

**Methods:**

**Measurement of ROS**

At 7 days after exposure to NG or HG, the constructs were washed in HEPES buffer saline solution (HBSS), pH 7.4 and incubated with 20μM DCFH-DA (30min at 37°C). Upon incubation, the constructs were enzymatically digested to isolate the cells from constructs. The DCF fluorescence of cells from 3D constructs was measured at 529nm with an excitatory wavelength of 495nm in a 96-well microplate reader (GENios, Tecan). ROS were expressed as DCF (relative fluorescence units)/ cell DNA (as evaluated by Hoechst staining).

**Supplementary Table 1**

Baseline characteristics and laboratory parameters of the three patients with calcific aortic valve stenosis from which the valvular cells were isolated.

| **Variables** | **Patient 1** | **Patient 2** | **Patient 3** |
| --- | --- | --- | --- |
| Sex | Male | Male | Male |
| Age, years | 74 | 64 | 71 |
| Glycemia mg/dl | 108 | 102 | 125 |
| Hypertension | No | Yes | Yes |
| Smoking, n (%) | No | No | No |
| HDL-C, mg/dl* | 20.9 | 43.2 | 41 |
| LDL-C, mg/dl* | 149.3 | 162.5 | 157 |
| ALP – alkaline phosphatase (U/L) | 154 | 161 | 140 |

* values measured under statin medication

**Supplementary figure 1**


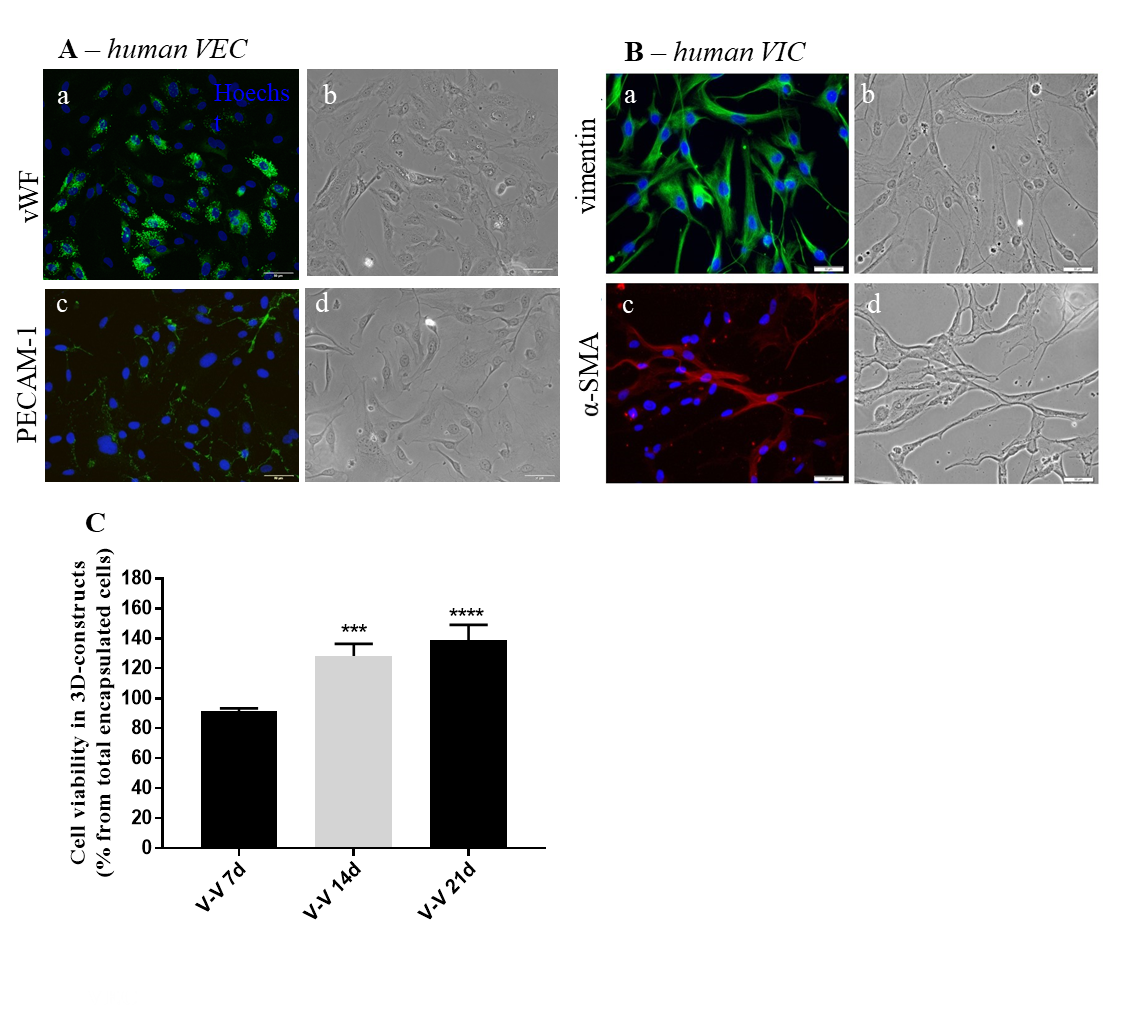


**Supplementary figure 2**

**
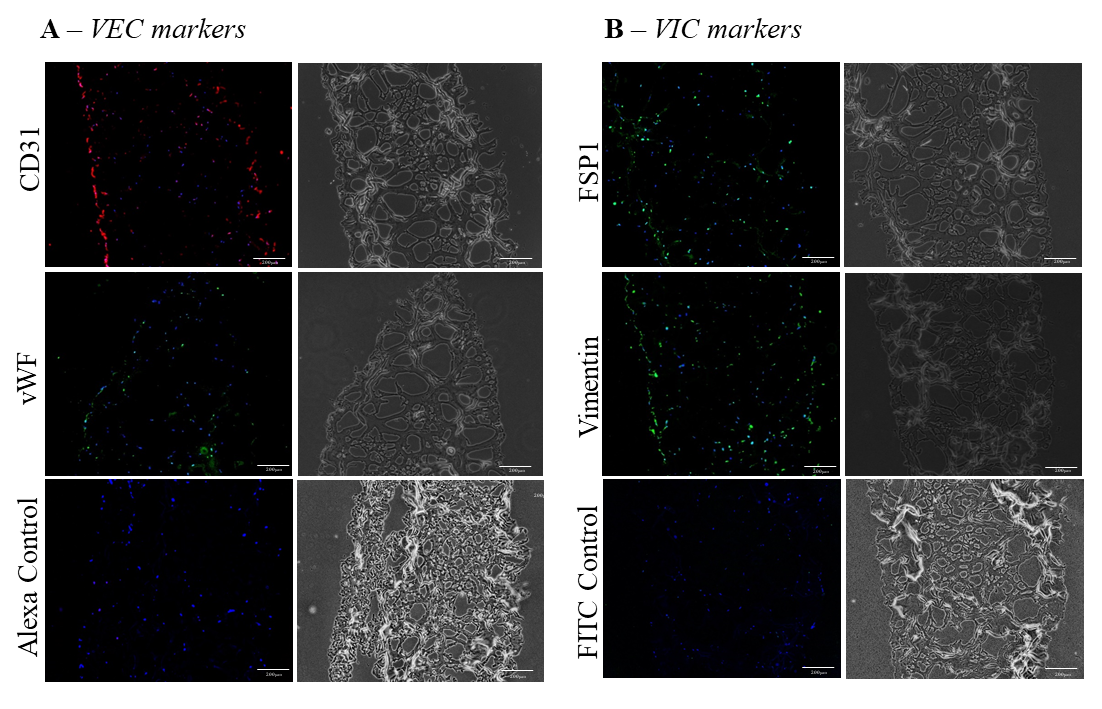
**

**Supplementary figure 3**

**Legends of supplementary figures:**

**Supplementary figure 1: *Expression of valve endothelial (A) and interstitial (B) cells specific markers in 2D culture****.* **A.** Positive fluorescent staining for (a) von Willebrand Factor and (c) CD31(PECAM-1) in the human aortic valve endothelial cells isolated from human operated valve. **B*.*** Positive fluorescent staining for (a) vimentin and (c) alpha-smooth muscle actin (α-SMA) of human aortic valve interstitial cells. DAPI was used as a counterstain; b, d– contrast phase images; scale bar=50 µm.**C.** Percentage (%) of live cells as quantified by Live/Dead cytotoxicity assay after 7, 14 and 21 days of culture of 3D constructs. The symbols ***p<0.001 ****p<0.0001 indicate significant changes of cell number at 14 and 21 days compared with 7days in 3D - constructs with VEC and VIC.

**Supplementary figure 2**. ***VEC and VIC markers in 3D constructs***. Immunofluorescence staining for VEC (A) and VIC (B) markers in 3D constructs with encapsulated VICs inside and VECs cultured on the surface (transversal sections), at 14 days of culture. Blue indicates nuclei (DAPI); Red indicates CD31, green indicates FSP-1, vimentin and vWF, as specified on every fluorescent image. Alexa and FITC controls represent section stained only with the secondary fluorescent antibody. All scale bars indicate 200 μm.

**Supplementary figure 3**. ***Effect of HG on the production of ROS in valvular cells from constructs: only with VEC (VEC, VECHG), only with VIC (VIC, VICHG) and with VEC and VIC (V-V, V-V HG).*** The ROS were determined by measuring the fluorescence of DCF-AM related to the total number of nuclei. The levels of reactive oxygen species are increased in cells from constructs exposed to 25mM glucose as compared to normal glucose (5mM), with a significant result in VIC (⁎p<0.05, n=2 experiments, with 2 constructs per experiment).
